# Neuraxial anesthesia for postpartum tubal ligation at an academic medical center

**DOI:** 10.12688/f1000research.16025.1

**Published:** 2018-09-26

**Authors:** Carlos Delgado, Wil Van Cleve, Christopher Kent, Emily Dinges, Laurent A. Bollag

**Affiliations:** 1 Department of Anesthesiology & Pain Medicine, University of Washington, Seattle, Seattle, Washington, 98195, USA

**Keywords:** obstetrical anesthesia, spinal anesthesia, epidural anesthesia, general anesthesia, tubal sterilization, postpartum period

## Abstract

**Background: **Use of an *in situ* epidural catheter has been suggested to be efficient to provide anesthesia for postpartum tubal ligation (PPTL). Reported epidural reactivation success rates vary from 74% to 92%. Predictors for reactivation failure include poor patient satisfaction with labor analgesia, increased delivery-to-reactivation time and the need for top-ups during labor. Some have suggested that this high failure rate precludes leaving the catheter *in situ* after delivery for subsequent reactivation attempts. In this study, we sought to evaluate the success rate of neuraxial techniques for PPTL and to determine if predictors of failure can be identified.

**Methods: **After obtaining IRB approval, a retrospective chart review of patients undergoing PPTL after vaginal delivery from July 2010 to July 2016 was conducted using CPT codes, yielding 93 records for analysis. Demographic, obstetric and anesthetic data (labor analgesia administration, length of epidural catheter in epidural space, top-up requirements, time of catheter reactivation, final anesthetic technique and corresponding doses for spinal and epidural anesthesia) were obtained.

**Results:** A total of 70 patients received labor neuraxial analgesia. Reactivation was attempted in 33 with a success rate of 66.7%. Patient height, epidural volume of local anesthetic and administered fentanyl dose were lower in the group that failed reactivation. Overall, spinal anesthesia was performed in 60 patients, with a success rate of 80%.

**Conclusions:** Our observed rate of successful postpartum epidural reactivation for tubal ligation was lower than the range reported in the literature. Our success rates for both spinal anesthesia and epidural reactivation for PPTL were lower than the generally accepted rates of successful epidural and spinal anesthesia for cesarean delivery. This gap may reflect a lower level of motivation on behalf of both the patients and anesthesia providers to tolerate “imperfect” neuraxial anesthesia once fetal considerations are removed.

## Introduction

Tubal ligation in the immediate postpartum period, postpartum tubal ligation (PPTL), is typically performed on the labor ward, but less than 50% of women that desire PPTL receive the procedure in the immediate postpartum period, despite The American College of Obstetricians and Gynecologists (ACOG) defining PPTL as an urgent procedure due to the limited optimal surgical timeframe ^1^. This gap between patient preference and observed outcome emphasizes the importance of an evidence-based anesthetic approach for PPTL to reduce barriers to receiving anesthetic care in the early postpartum period. Use of an existing labor epidural catheter has been proposed as an efficient way to provide anesthesia for PPTL ^2^. Older studies reported epidural reactivation success rates varying from 74% to 92% ^2– 4^. Recently, Powell *et al*. reported an epidural reactivation success rate of 78% in a prospective observational study of anesthesia for PPTL and outlined the risk factors for failed reactivation of an epidural catheter. Predictors of failure included poor patient satisfaction with labor analgesia, increased delivery-to-reactivation time, and the need for manual top-ups during labor and delivery ^5^. Within our practice, and sometimes in the broader obstetric anesthesia community, providers have suggested that rates of failure when attempting catheter “reactivation” for PPTL do not support the practice leaving a labor epidural catheter in place for an interval PPTL. Instead, these providers advocate the routine removal of the epidural catheter followed by a *de novo* spinal anesthetic (SA). This study evaluated the frequency of success at our center using the aforementioned anesthetic techniques for PPTL and sought to determine if there are clinical success predictors that can aid in anesthetic decision-making.

## Methods

### Ethical approval

This retrospective observational study was approved by the University of Washington Review Board, which waived the requirement of informed consent (HSD Study STUDY0000117). A chart review of all medical records in the labor and delivery unit from July 2010 to July 2016 was conducted to identify patients with CPT codes for bilateral tubal ligation that occurred consecutively after vaginal delivery. No exclusion criteria were applied.

### Data collection

Data collected for each case included demographic data (age, body mass index), obstetric data (gravidity, parity, gestational age achieved) and anesthetic data (type of labor analgesia: combined-spinal epidural or straight lumbar epidural; number of regional anesthesia attempts performed; length of epidural catheter in epidural space, top-up requirements during labor, time of catheter reactivation after delivery, if applicable; and the initial and final anesthetic techniques used to complete the case: successful epidural reactivation, *de novo* spinal anesthetic or general anesthesia). Perioperative doses for medications for spinal and epidural anesthetics as well as the use of supplemental sedative/hypnotic agents were also collected. Successful epidural reactivation was defined as completion of the surgical procedure under epidural analgesia.

### Data analysis

Statistical analysis of collected data was performed using R version 3.4.3 (R Foundation for Statistical Computing, Vienna, Austria). Univariate distributions are described as proportions, means and standard deviations, or medians and interquartile ranges, as appropriate. Continuous variables were compared using t-tests and ordinal variables were compared using Fisher’s exact test. Statistical significance was pre-specified as p < 0.05.

## Results

Data from 93 patients were analyzed. Neuraxial analgesia for labor was used in 70 patients (75%). Of these patients that received labor analgesia, 33 (47%) underwent attempts at reactivation, with a success rate of 66.7% (22 patients). For this group of patients, the mean documented length of catheter in space was 4.9 (± 0.3) cm. A total of four patients (18%) required top-ups during labor. Median time to reactivation after delivery was 4.8 (IQR 3.3–9.8) hours. The mean volume of local anesthetic used to initiate anesthesia for the surgical procedure was 21.7 (± 7.6) ml. The mean epidural fentanyl dose was 86.7 (± 22.9) µg. Intravenous midazolam (1.9 ± 0.5 mg) and fentanyl (68.2 ± 35.5 µg) were also given in 16 patients. When comparing the characteristics of successful and unsuccessful epidural reactivations, we observed that patient height (163 ± 7.2 cm versus 158 ± 6.0 cm, p = 0.03), volume of local anesthetic administered during reactivation (21.7 ± 7.6 ml versus 14.8 ± 13.2 ml, p = 0.03), and dose of epidural fentanyl (86.7 ± 22.9 µg versus 63 ± 24.4 mcg, p = 0.03) were lower in the group that failed catheter reactivation. Total intravenous fentanyl was also higher (127.1 ± 57.6 µg) in this group compared to the successful group (68.2 ± 35.5 µg) (p = 0.007) ( Table 1).

**Table 1.** Demographic, obstetric and anesthetic data in patients in which epidural reactivation for postpartum tubal ligation was attempted.

Variables	Successful reactivation (n = 22)	Failed reactivation (n = 11)	P value
Age (years)	31.3 ± 4.4	30 ± 3.5	0.21
Height (cm)	*163 ± 7.2*	*158 ± 6.0*	*0.03*
BMI	33 ± 6.0	31.4 ± 5.1	0.24
Gestational age (weeks)	37.9 ± 2.4	37.4 ± 3.0	0.32
Length of catheter in space (cm)	4.9 ± 0.4	4.9 ± 1.0	0.48
Patients requiring top-ups during labor (n, %)	4, 18.1%	5, 45%	0.09
Duration of epidural analgesia (h), IQR (h)	4.3, 2.3–6.7	3.9, 1.8–5.4	0.47
Time to reactivation (h), IQR (h)	4.8, 3.3–9.8	4, 1.9–11.2	0.38
Total epidural local anesthetic (ml)	*21.7 ± 7.6*	*14.8 ± 13.2*	*0.03*
Total epidural fentanyl (µg)	*86.7 ± 22.9*	*63 ± 24.4*	*0.03*
Total IV midazolam (mg)	1.9 ± 0.5	1.8 ± 0.3	0.48
Total IV fentanyl (mg)	*68.2 ± 35.5*	*127.1 ± 57.6*	*0.007*

All data presented as mean ± standard deviation; median, interquartile range; percentage. T-test and Fisher’s exact test, p < 0.05 for statistical significance. BMI, body mass index; IQR, interquartile range; IV, intravenous.

In patients in which reactivation was unsuccessful, a rescue SA was attempted in five cases with a success rate of 80%. The patient that failed SA (bupivacaine 11.2 mg, no opioid) had received large amounts of epidural solution at reactivation (30 ml chloroprocaine 3% and 10 ml lidocaine 2%). General anesthesia was the final anesthetic technique for the remaining unsuccessful epidural reactivations and the failed SA.

Epidural reactivation was not attempted in 37 patients (53%). There were two cases that received general anesthesia as the primary technique. One patient received combined spinal epidural (CSE) anesthesia as the primary technique due to maternal congenital cardiac disease. SA was performed after removal of epidural catheter in 34 patients. This technique was successful in 25 patients (74%). For the cases in which SA block was not achieved, general anesthesia (8 patients) or CSE anesthesia (1 patient) were used.

Single-shot spinal anesthesia in patients with pre-existing epidural catheters (combining those in whom epidural reactivation was and was not attempted) was performed in 39 patients, with an overall success rate of 74%. An attempt to reactivate the catheter prior to spinal placement had been carried out in 4 patients (13.8%), with a median elapsed time of 3.3 (IQR 0.7–9.6) hours after delivery and an average volume of 12.7 ± 8.6 ml of local anesthetic. Due to insufficient levels of anesthetic blockade after attempt at reactivation, SA was chosen as the rescue anesthetic technique. The mean volume of local anesthetic (hyperbaric bupivacaine 0.75%) was 1.5 ± 0.3 ml. The mean intrathecal fentanyl dose was 14.2 ± 6.4 µg.

Data regarding demographic, obstetric and anesthetic variables comparing successful versus unsuccessful SA in patients with a pre-existing epidural catheter is presented in Table 2. Apart from a more advanced gestational age in the failed SA group, no statistical differences existed between successful and unsuccessful SAs.

**Table 2.** Demographic, obstetric and anesthetic data in patients with a pre-existing epidural catheter in which spinal anesthesia (SA) was attempted for postpartum tubal ligation.

Variable	Successful SA (n = 29)	Failed SA (n = 10)	P value
Age (years)	32.2 ± 4.7	32.7 ± 5.5	0.39
Height (cm)	165 ± 8.2	163 ± 6.8	0.25
BMI	30.8 ± 4.6	30.7 ± 7.6	0.48
Gestational age (weeks)	*36 ± 4.1*	*39 ± 0.7*	*0.02*
Length of catheter in space (cm)	4.9 ± 0.6	4.6 ± 0.4	0.11
Patients requiring top-ups during labor (n, %)	13, 45%	3, 33%	0.41
Duration of epidural analgesia (h), IQR (h)	5.5, 2.4–8.4	2.6, 1.3–8.5	0.44
Epidural reactivation attempted (n, %)	4, 13.8%	1, 10%	0.75
Time to reactivation (h), IQR (h)	3.3, 0.7 – 9.6	12.8 *	NA
Total epidural local anesthetic (ml)	12.7 ± 8.6	40 *	NA
Total intrathecal local anesthetic (ml)	1.5 ± 0.3	1.5 ± 0.2	0.27
Total intrathecal fentanyl (µg)	14.2 ± 6.4	18 ± 7.6	0.12
Total IV midazolam (mg)	1.9 ± 0.6	2.3 ± 2.1	0.24
Total IV fentanyl (mg)	96 ± 59.2	120.7 ± 52.6	0.16

All data presented as mean ± standard deviation; median, interquartile range; percentage. T-test and Fisher’s exact test, p < 0.05 for statistical significance. *Only one patient in this group underwent reactivation. No statistical calculations were performed. BMI, body mass index; IQR, interquartile range; IV, intravenous; NA, not applicable.

Of the 23 patients who did not have a pre-existing epidural catheter at the time of PPTL, 21 (91%) received a single-shot spinal block, with a success rate of 91%. The remaining two cases were performed under general anesthetic and with CSE as initial technique. The mean volume of local anesthetic (hyperbaric bupivacaine 0.75%) was 1.5 ± 0.2 ml. The mean intrathecal fentanyl dose was 15.2 ± 6.8 µg. Intravenous midazolam (mean 2.1 ± 0.9 mg) and fentanyl (mean 87.5 ± 59.4 µg) were also given as adjuvants. No statistical analysis was performed to compare success at performing SA in patients without pre-existing epidurals given the high success rate of this technique.

A review of all cases of attempts at SA (patients with a prior epidural catheter, irrespective of attempts at reactivation, and patients without a pre-existing epidural catheter) revealed a spinal block success rate of 80% (48 of 60 cases). Mean intrathecal doses were 1.5 ± 0.2 ml of hyperbaric bupivacaine 0.75% and 14.2 ± 6.2 µg of fentanyl. Intrathecal fentanyl doses above 20 µg added to bupivacaine were associated with spinal failure (p = 0.001). No other demographic, obstetric or anesthetic factors were statistically different ( Table 3).

**Table 3.** Demographic, obstetric and anesthetic data in patients in which spinal anesthesia (SA) was attempted for postpartum tubal liugation (overall).

Variable	Successful SA (n = 48)	Failed SA (n = 12)	P value
Age (years)	32.5 ± 5.0	32.5 ± 5.8	0.47
Height (cm)	164 ± 7.0	162 ± 6.7	0.29
BMI	30.4 ± 4.1	30.3 ± 7.3	0.47
Gestational age (weeks)	*37 ± 3.6*	*39 ± 0.7*	*0.05*
History of epidural catheter placement (n, %)	29, 60%	10, 83%	0.13
Total intrathecal local anesthetic (ml)	1.5 ± 0.2	1.5 ± 0.2	0.32
Total intrathecal fentanyl (µg)	*14.2 ± 6.2*	*20 ± 6.5*	*0.01*

All data presented as mean ± standard deviation; median; percentage. T-test and Fisher’s exact test, p < 0.05 for statistical significance. BMI, body mass index.

**Figure 1.**
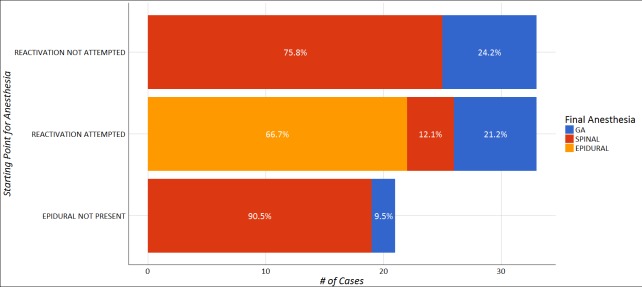
Distribution of final anesthetic technique for postpartum tubal ligation in relation to success of the initial technique.

The final distribution of anesthetic technique and success used for the PPTL is presented in Figure 1.

Complete data on demographics and the treatment given to each patient surrounding postpartum tubal ligation, including details on treatment method and the pharmaceuticals used (with dose) Also included is a guide to the abbreviations used. Click here for additional data file. Copyright: © 2018 Delgado C et al. 2018 Data associated with the article are available under the terms of the Creative Commons Zero "No rights reserved" data waiver (CC0 1.0 Public domain dedication).

## Discussion

In a review of 6 years of data from our practice, we observed a success rate of 67% when attempting to use *in situ* epidural catheters for PPTL, lower than we expected given the published literature on this topic. A recent retrospective review (n = 202) of PPTL anesthesia reported an epidural reactivation success rate of 74% ^6^. A prospective study (n = 100) designed to assess the risk factors for failed epidural reactivation reported a success rate of 78% ^5^. In analyzing our data, we found no association between previously noted risk factors and epidural failure. Vincent *et al*. reported that a shorter time interval between delivery and reactivation attempt was a predictor for success, with reinjection within 4 hours of delivery having the highest success rate ^3^. These findings are in agreement with data from the recent prospective study by Powell *et al*. ^5^. Others have suggested that the time interval between catheter insertion and reactivation is of importance, with a period less than 24 hours as a more reliable predictor of success compared to time between delivery and reactivation ^6^. Our findings do not reveal any relationship between time of catheter placement or delivery and success of reactivation. The majority of catheters were reactivated within 5 hours of delivery, while only one was used more than 12 hours after delivery. 

The immediate postpartum period is the ideal time to perform PPTL due to the ease and convenience for both physicians and patients ^1^. In fact, this procedure is defined by ACOG as an urgent procedure, because failure to accomplish the procedure during the same hospitalization as delivery may make the procedure more complex and increase the risk of unintended pregnancy in the first year following birth ^7, 8^. The availability of nursing, anesthesia, and obstetric staff for this procedure alongside busy workloads on the labor ward may contribute to failures in achieving a pre-discharge PPTL.

The probability of pain during surgery has been linked with the height of the patient under epidural anesthesia, but only in very short and tall patients, and it significantly interacts with weight ^9^. Although we observed that the mean height in the group that failed reactivation was lower (close to 5 cm), we struggle to find a biological plausibility to this finding. Administration of higher volumes of epidural solution increases dermatomal spread, particularly with bolus administration ^10^. The group in which reactivation was unsuccessful received a lower volume of both local anesthetic and opioid. Epidural medication is typically incrementally titrated during catheter reactivation. If, early during the reactivation attempt, an inadequate or patchy block is noted, epidural reactivation is typically aborted to avoid a potentially high rescue SA. This could potentially explain the lower volume of local anesthetic used in that group. While an increased use of fentanyl in the failed epidural reactivation group is observed, this corresponds to the total dose, which includes cases with conversion to general anesthesia.

For those without preexisting epidural analgesia, the successful use of spinal anesthesia with hyperbaric bupivacaine with or without opioids has been reported by multiple authors ^11, 12^. This technique was favored by most providers for patients who had labored without neuraxial analgesia with a high rate of success. Administration of a dose of local anesthetic similar to that used for cesarean deliveries (e.g. 12 mg of bupivacaine) seems to provide sufficient anesthesia for PPTL ^12, 13^.

When spinal anesthesia was attempted in patients with a history of epidural analgesia during labor, however, our success rate was found to be only 80%. This is a higher rate than the reported 2–6% range of failure described for spinal anesthesia for cesarean delivery ^14, 15^ and clearly higher than the 1% conversion rate of regional to general anesthesia due to failed spinal anesthesia, as recommended by the Royal College of Anesthetists ^16^. Spinal anesthesia failure was three times higher in women where an epidural was used but not topped up for emergent cesarean delivery in a large retrospective audit of over 5000 cesarean deliveries ^15^. Clear free flowing cerebrospinal fluid (CSF) is associated with a successful spinal block. Prior injection of local anesthetic into the epidural space could lead providers to mistake epidural local anesthetic return through a spinal needle for CSF, which might provide an explanation for failure to achieve SA after epidural analgesia had been performed ^17^.

Some evidence points to a need in increasing doses of local anesthetic used in SA for PPTL to adjust for changes in segmental blockade requirements in the postpartum ^18^. Huffnagle *et al*. found that while 7.5 mg of hyperbaric bupivacaine provided adequate surgical anesthesia for this procedure, some failed spinals occurred at this dose ^19^. The mean local anesthetic doses used in our institution were similar to our standard for cesarean deliveries (1.4 ml of hyperbaric bupivacaine 0.75%), and we did not find a difference between successful and failed SAs in our patients. We also found that advanced gestational age was linked to failure of SA, whether there had been any epidural space manipulation. Our finding contrasts with reports of inadequate surgical anesthesia for cesarean deliveries in pre-term parturients, even though it was determined that low fetal weight was the main factor implicated ^20^. Notably, fentanyl doses above 20 µg were observed overall in the spinal failure group, without being associated to a decrease in corresponding local anesthetic dosage.

Conversion to general anesthesia in the obstetric patient after neuraxial anesthesia placement is often the result of decreased patient tolerance to pain during the procedure, in addition to concerns by the surgical team, as reported by a large retrospective review of over 35,000 spinal anesthetics for cesarean delivery by Guglielmo *et al*. In this study, SA was impossible to perform in a few rare cases. More commonly, the block was achieved but was insufficient to provide adequate surgical block ^21^. General anesthesia was used in 55% of cases of PPTL in small community hospitals according to the Obstetric Anesthesia Workforce Survey; this contrasts with the use of general anesthesia in less than 25% of the cases of PPTL in large-center referral center hospitals affiliated with university programs ^22^. Ultimately, the decision to use a particular type of anesthetic for PPTL should be individualized based on obstetric and anesthetic factors, as well as taking into account patient preference. Regional anesthesia, however, seems to be the favored approach, as the time for maternal physiology to return baseline in the postpartum period is not well delineated ^23^.

There are several limitations to our study. As a single-institution retrospective study with small numbers of patients in the subgroups of interest and relatively few procedures, we can simply add to the existing studies on this topic with limited generalizability. Further, even though each record was personally reviewed by one of the authors (all of whom are members of the obstetric anesthesia division) to minimize the amount of missed data and erroneous coding regarding type of anesthesia, our data collection is potentially subject to bias due to anesthetic technique preferences on the part of the research team. Documentation of the reasons for favoring attempts at reactivation over proceeding with spinal anesthesia directly was not consistently found in the clinical records. In most of the cases in which reactivation was not attempted despite the presence of an epidural catheter that functioned well during labor and was left *in situ*, no rationale for this decision could be found in the medical records. Provider preference could have been guided by either a distrust of a catheter that has not been infused for some time or lack of patience or time available to reactivate the catheter to achieve adequate surgical anesthesia. Prior provider experience with failed epidurals might also have played a part. Some have recommended spinal anesthesia even in parturients with indwelling epidural catheters to avoid less-than-perfect epidural reactivation rates and minimize time delays and costs ^4^. In fact, in a published survey of BTL practices in academic institutions, up to 40% of respondents elect not to leave a catheter in situ to be used after delivery ^6^. Our study is obviously not powered to evaluate complication rates associated with the different anesthetic techniques used for PPTL. One of the largest studies of PPTL found very low rates of complications of any type and, notably, 86% of the procedures in this series were done with general anesthesia ^24^.

In summary, our study found lower success rates for epidural reactivation for PPTL than those reported in the literature. There was also a lower success rate for spinal anesthetics placed after an epidural catheter was used to provide labor analgesia. We were unable to find clinical predictors for the failure rate. The need for conversion to general anesthesia, besides being attributed to an insufficient block, may reflect a lower level of motivation on behalf of both the patients and anesthesia providers to tolerate suboptimal anesthesia when fetal considerations are no longer a factor and some aspects of maternal physiology are already less concerning for the use of general anesthesia.

## Data availability

The data referenced by this article are under copyright with the following copyright statement: Copyright: © 2018 Delgado C et al.

Data associated with the article are available under the terms of the Creative Commons Zero "No rights reserved" data waiver (CC0 1.0 Public domain dedication). 

**Dataset 1. Complete data on demographics and the treatment given to each patient surrounding postpartum tubal ligation, including details on treatment method and the pharmaceuticals used (with dose).** Also included is a guide to the abbreviations used. DOI: https://doi.org/10.5256/f1000research.16025.d218466 ^25^.
