# Supplementary material for: Neuraxial anesthesia for postpartum tubal ligation at an academic medical center
Source: F1000Res. 2018 Sep 26;7:1557. [Version 1] doi: 10.12688/f1000research.16025.1 (PMC6305207; doi:10.12688/f1000research.16025.1)
Supplement: Complete data on demographics and the treatment given to each patient surrounding postpartum tubal ligation, including details on treatment method and the pharmaceuticals used (with dose) — Also included is a guide to the abbreviations used. [file f1000research-7-17502-s0000.tgz › 07baf0ad-a5b5-4eba-829c-1b782865d8cc_Data.zip!/Data/PPTL_definition_variables.docx]

***PPTL_mystats***

***Definition of Variables***

**Age =** age in years

**Weight =** weight in kilos, no decimals, if present, round up to next whole number

**Height =** height in cm, no decimals.

**BMI (body mass index)=** value

**Gravidity =** number of times patient has been pregnant

**Parity =** number of times pregnancy has reach a viable gestational age and has been delivered (including stillbirths and live births)

**Gestational Age =** on weeks, no decimals

**Labor Epidural? =** 1 YES, 2 NO

**CSE (combined spinal-epidural) =** 1 YES, 2 NO

**EPID (epidural) =** 1 YES, 2 NO

**Date and Time of placement =** of neuraxial analgesia technique, month/day/year and 00:00 (24h)

**#Attempts =** total number of attempts for successful placement

**LOR (loss of resistance) =** distance to loss of resistance (with saline) in (cm)

**Catheter distance at skin** = in (cm)

**# Top-ups =** total number for the duration of labor

**Date and Time of analgesia end time =** month/day/year and 00:00 (24h)

**Length epidural analgesia =** duration of epidural analgesia in (min)

**Catheter reactivation attempted? =** 1 YES, 2 NO

**Catheter reactivation date and time =** first dose local anesthetic in operating room (OR), even outside if given by any provider in this manner; month/day/year and 00:00 (24h)

**Catheter reactivation successful? =** 1 YES, 2 NO

**Elapsed time until activation =** time between analgesia end time and catheter reactivation, in (min)

**Anesthesia start time =** time to arrive to OR, 00:00 (24h)

**Final Type of Anesthesia =** 1 for GENERAL, 2 for EPIDURAL, 3 SPINAL

**If SPINAL, successful?** = 1 YES, 2 NO

**Surgery start time =** 00:00 (24h)

**Surgery stop time =** 00:00 (24h)

**Type of local anesthetic =** 1 for LIDOCAINE, 2 for CHLOROPROCAINE, 3 for BUPIVACAINE

**Total local anesthetic =** total dose in mL

**Fentanyl IV =** total intravenous dose in (mcg)

**Fentanyl EPID =** total epidural dose in (mcg)

**Fentanyl SPINAL =** total spinal (intrathecal) dose in (mcg)

**Midazolam =** total dose in (mg)

**Ketamine =** total dose in (mg)
