# Supplementary material for: Neuraxial anesthesia for postpartum tubal ligation at an academic medical center
Source: F1000Res. 2018 Sep 26;7:1557. [Version 1] doi: 10.12688/f1000research.16025.1 (PMC6305207; doi:10.12688/f1000research.16025.1)
Supplement: Peer review history

# Peer review: Referee response for version 1

**DOI:** 10.5256/f1000research.17502.r40801

**Published:** 2019-02-04

**Authors:** Feyce Peralta

**Affiliations:** 1 Department of Anesthesiology, Feinberg School of Medicine, Northwestern University, Chicago, IL, USA

**General comments: **Relevant topic for obstetricians and obstetric anesthesiologists with key public health implications. Authors evaluated success rate of different neuraxial techniques for PPTL. Retrospective study using small dataset from a single institution.

**Introduction: **“Tubal ligation in the immediate postpartum period, postpartum tubal ligation (PPTL), is typically performed on the labor ward, but less than 50% of women that desire PPTL receive the procedure in the immediate postpartum period, despite The American College of Obstetricians and Gynecologists (ACOG) defining PPTL as an urgent procedure due to the limited optimal surgical timeframe1.” I suggest placing reference number 8 (Thurman) before current reference number 1 (Committee opinion no. 530). Consider splitting this sentence in two.

**Methods: **Retrospective study of patients that underwent PPTL after vaginal delivery over a 6-year period at a single institution.

- I recommend expanding the definition of successful epidural catheter reactivation (e.g., completion of PPTL under epidural **anesthesia **with same epidural catheter previously used for labor analgesia and delivery).
- The authors should clarify who performed the chart review. 1 investigator? 2 investigators?
- The authors could include a pregnancy risk stratification (low vs. high risk) as part of their demographic data. Albanese *et al.* (2017 ^1^) reported on the request and fulfilment of PPTL according to pregnancy risk. Anesthetic practices are likely to be affected by this variable.

**Results: **The authors analyzed data from a very small number of patients. Overall, this section is very easy to read and to follow. 

**Discussion: **The greatest limitation of this study is the small sample size. Therefore, conclusions cannot be made based on this study regarding predictors for the success of neuraxial anesthesia for PPTL. Future directions could be added to the discussion section.

- The authors should consider moving the second and third sentences to the introduction section.

I have read this submission. I believe that I have an appropriate level of expertise to confirm that it is of an acceptable scientific standard.

## References

[1] AlbaneseAFrenchMGossettDR: Request and fulfillment of postpartum tubal ligation in patients after high-risk pregnancy. *Contraception*.2017;95(3) : 10.1016/j.contraception.2016.08.009 234-238 10.1016/j.contraception.2016.08.009 27570140

# Peer review: Referee response for version 1

**DOI:** 10.5256/f1000research.17502.r40803

**Published:** 2018-12-12

**Authors:** Dana R. Gossett

**Affiliations:** 1 Department of Obstetrics, Gynecology and Reproductive Sciences, University of California, San Francisco (UCSF), San Francisco, CA, USA

General comments:

 This is a topic of interest to obstetric anesthesiologists and obstetricians, and highlights the role of obstetric anesthesiologists in providing access to a needed service that often goes unprovided (postpartum tubal ligation—PPTL). The authors examine success rates of pre-existing labor epidurals for surgical anesthesia for tubal ligation.

Study design:

 This is a retrospective study of undergoing postpartum tubal ligation at a single institution over a 6 year period. Method of labor analgesia and method of anesthesia for the PPTL were collected, and rate of successful re-use of the labor epidural for the PPTL was calculated.

Abstract:

 The abstract provides an accurate synopsis of the paper’s design and findings.

Introduction:

 Overall the introduction is quite short, but does provide appropriate background and citations. Do the authors have a citation to support the statements “Within our practice, and sometimes in the broader obstetric anesthesia community, providers have suggested that rates of failure when attempting catheter “reactivation” for PPTL do not support the practice leaving a labor epidural catheter in place for an interval PPTL. Instead, these providers advocate the routine removal of the epidural catheter followed by a de novo spinal anesthetic (SA)”?

Materials & Methods:

 This is a retrospective review of all women undergoing PPTL over a 6 year period. Data were collected regarding demographic and obstetric factors, anesthetic method and dosing, and factors previously identified as predictors of failure of epidural “reactivation” after delivery.

Results, Tables/Figures:

 In general, the results are clearly communicated. During the study period, only 93 women undergo PPTL, 70 of whom have labor epidurals. Of these, only 33 actually have an attempt to reactivate the epidural for use during the PPTL—this then represents the target population, a very small number. Of these, 67% had successful reactivation of their epidurals.

 For women who received spinal anesthesia (SA) to complete their PPTL, fewer had successful SA if they’d had a prior epidural (74% successful spinal vs 91% of those who had not had a labor epidural.)

Discussion:

 The discussion section is very long in proportion to the remainder of this paper and could be substantially trimmed. Paragraph 2 could be moved to the introduction/background.

 While the authors state that previously published predictors of epidural re-activation were not confirmed in this study, they are underpowered to evaluate some. For example, the number of patients requiring “top ups” (redoses of the epidural) was 9, with 4 in the successful reactivation group and 5 in the failed group—for rates of 18% and 45%. Because of small numbers, the p-value for this comparison is 0.09, but it is likely that it would reach statistical significance with greater numbers.

 One of the most significant limitations, as acknowledged by the authors, is the lack of information about why/how decisions were made by the anesthesiologists about whether or not to use an existing epidural, and what to use next or instead. This introduces the potential for selection bias of subjects having re-activation attempts.

 It would be ideal if the authors concluded their discussion by tying their findings back to the larger question they are investigating—can they make any recommendations about what the optimal method would be? Or, as their study may not provide this, what type of investigation would they recommend to get to that answer?

Summary:

 This retrospective study of anesthetic use for PPTL demonstrated a lower rate of successful re-activation of labor epidurals than previous reports. There is no clear explanation of this unanticipated finding.

 A prospective trial would better address this by removing clinician bias/practice patterns from the decision-making about anesthetic mode, and limiting differences (known and unknown) between the two groups of patients. Given the infrequency of PPTL, a multicenter trial would be required to accrue patients in a reasonable amount of time, so the choice of a retrospective review is understandable.

I have read this submission. I believe that I have an appropriate level of expertise to confirm that it is of an acceptable scientific standard.

# Peer review: Referee response for version 1

**DOI:** 10.5256/f1000research.17502.r38781

**Published:** 2018-10-18

**Authors:** Christine P. McKenzie

**Affiliations:** 1 Department of Anesthesiology, University of North Carolina Medical Center (UNC Medical Center), Chapel Hill, NC, USA

The article presents a small retrospective review of anesthetic management for postpartum tubal ligations. The article is well-written and organized. The data are presented clearly and concisely. 

 The author outlines limitations of the study such as a small number of patients and generalizability. However, it adds to the current literature on PPTL and higher than expected neuraxial anesthesia failure rates. There are no identified predictors for success/failure of epidural reactivation; however, given the low numbers the study is underpowered to access predictors. 

 The article discusses the clinical considerations for anesthetic management and the importance of completing these urgent PPTL procedures.

I have read this submission. I believe that I have an appropriate level of expertise to confirm that it is of an acceptable scientific standard.
